# Biosynthesis of methylated resveratrol analogs through the construction of an artificial biosynthetic pathway in *E. coli*

**DOI:** 10.1186/1472-6750-14-67

**Published:** 2014-07-17

**Authors:** Sun-Young Kang, Jae Kyoung Lee, Oksik Choi, Cha Young Kim, Jae-Hyuk Jang, Bang Yeon Hwang, Young-Soo Hong

**Affiliations:** 1 Chemical Biology Research Center, Korea Research Institute of Bioscience and Biotechnology(KRIBB), 30 Yeongudanji-ro, Ochang-eup, Chungbuk 363-883, Republic of Korea; 2 Department of Pharmacy Graduate School, Chungbuk National University, Cheongju 361-763, Republic of Korea; 3 Eco-friendly Bio-Material Research Center, KRIBB, Jeongeup 580-1853, Republic of Korea

## Abstract

**Background:**

Methylated resveratrol analogs show similar biological activities that are comparable with those of the resveratrol. However, the methylated resveratrol analogs exhibit better bioavailability as they are more easily transported into the cell and more resistant to degradation. Although these compounds are widely used in human health care and in industrial materials, at present they are mainly obtained by extraction from raw plant sources. Accordingly their production can suffer from a variety of economic problems, including low levels of productivity and/or heterogeneous quality. On this backdrop, large-scale production of plant metabolites via microbial approaches is a promising alternative to chemical synthesis and extraction from plant sources.

**Results:**

An *Escherichia coli* system containing an artificial biosynthetic pathway that produces methylated resveratrol analogues, such as pinostilbene (3,4’-dihydroxy-5-methoxystilbene), 3,5-dihydroxy-4’-methoxystilbene, 3,4’-dimethoxy-5-hydroxystilbene, and 3,5,4’-trimethoxystilbene, from simple carbon sources is developed. These artificial biosynthetic pathways contain a series of codon-optimized *O*-methyltransferase genes from sorghum in addition to the resveratrol biosynthetic genes. The *E. coli* cells that harbor pET-opTLO1S or pET-opTLO3S produce the one-methyl resveratrol analogues of 3,5-dihydroxy-4’-methoxystilbene and pinostilbene, respectively. Furthermore, the *E. coli* cells that harbor pET-opTLO13S produce 3,5-dihydroxy-4’-methoxystilbene, *bis*-methyl resveratrol (3,4’-dimethoxy-5-hydroxystilbene), and *tri*-methyl resveratrol (3,5,4’-trimethoxystilbene).

**Conclusions:**

Our strategy demonstrates the first harness microorganisms for *de novo* synthesis of methylated resveratrol analogs used a single vector system joined with resveratrol biosynthetic genes and sorghum two resveratrol *O*-methyltransferase genes. Thus, this is also the first report on the production of the methylated resveratrol compounds *bis*-methyl and *tri*-methyl resveratrol (3,4’-dimethoxy-5-hydroxystilbene and 3,5,4’-trimethoxystilbene) in the *E. coli* culture. Thus, the production of the methylated resveratrol compounds was performed on the simple *E. coli* medium without precursor feeding in the culture.

## Background

Resveratrol (3,5,4’-trihydroxystilbene), which is found in red wine and grapes as well as in other plants, has been the subject of intensive studies that focus on its possible role in preventing cardiovascular heart diseases and cancer [1]. Resveratrol and its analogs have important functions as antimicrobial and antioxidant compounds in plant defense responses to environmental stresses, such as UV irradiation and fungal infection. However, they also exhibit diverse beneficial properties in humans, including anti-inflammatory effects, anti-tumor activities, and anti-aging effects [2]. In particular, over the past decade, resveratrol has been used as a dietary supplement that has been demonstrated to possess a broad spectrum of pharmacological properties [3,4]. However, Walle *et al*. reported the high absorption but rapid metabolism of resveratrol when it was administered orally to humans [5]. Several reports have demonstrated that the methylation of resveratrol results in the enhancement of its bioavailability and bioactivity [6,7]. For example, pterostilbene is a 3,5 *bis*-methylated resveratrol that is present in blueberries and it has been investigated extensively [8]. The substitution of the hydroxy with the methoxy groups increases the lipophilicity of pterostilbene over the resveratrol, which results in high bioavailability. These differences in the pharmacokinetics might explain the higher biological activity of pterostilbene over its parental compound resveratrol [9]. Furthermore, 3,5,4’-trimethoxystilbene has emerged as the most potent proapoptotic analog of resveratrol [10]. In addition, 3,5,4’-trimethoxystilbene and pinostilbene (3,4’-dihydroxy-5-methoxystilbene) were reported to be up to 100-fold more cytotoxic than resveratrol in cancer cell lines [11]. Consequently, methylated resveratrol analogs, which can possess greater oral bioavailability due to their decreased metabolism and increased absorption, have garnered increasing attention as alternative chemopreventive agents. Therefore, methylated resveratrols have become an attractive target for bioengineering, but few attempts to characterize the methylation enzyme of resveratrol have been reported thus far [12-17].

**Figure 1:**
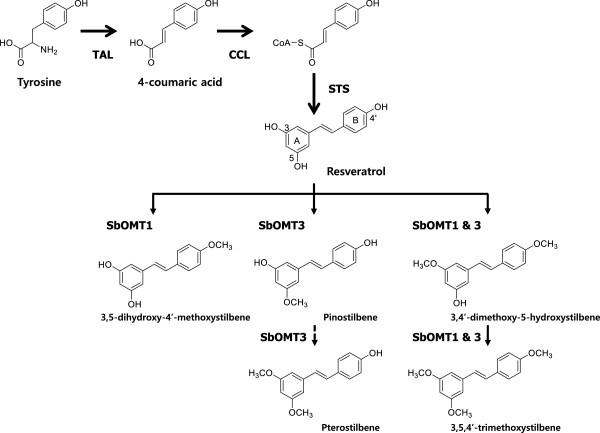
**Engineered biosynthetic pathways for the methylated resveratrol analogs starting from tyrosine in *E. coli*.** TAL (tyrosine ammonia-lyase) from *S. espanaensis*, CCL (4-coumarate:CoA ligase) from *S. coelicolor*, STS (stilbene synthases) from *A. hypogaea*, and sbOMT1 & sbOMT3 (resveratrol *O*-methyltransferases) from *S. bicolor*.

Methylated resveratrol is biosynthesized from the general phenylpropanoid pathway beginning with phenylalanine or tyrosine, or both. The methylated resveratrol biosynthesis pathway designed for use in this study is depicted in Figure 1. In this pathway, tyrosine is converted into 4-coumaric acid using tyrosine ammonia lyase (TAL). The 4-coumaric acid is then activated to 4-coumaroyl-CoA using the 4-coumarate-CoA ligase (4CL). This 4-coumaroyl-CoA is condensed with three molecules of malonyl-CoA via stilbene synthase (STS), which is the key enzyme in resveratrol synthesis. Resveratrol is converted into its methylated derivatives through the resveratrol *O*-methyltransferase. Meanwhile, several attempts to produce resveratrol using recombinant microorganisms, such as *Escherichia coli*[18-23] and *Saccharomyces cerevisiae*[24,25], have been reported. However, little has been reported about the characterization of the resveratrol *O*-methyltransferase function in plants [13-15,17]. Recently, the *sbOMT1* and *sbOMT3* genes from *Sorghum bicolor* were reported and their functions were characterized as different resveratrol *O*-methyltransferases (ROMTs), respectively [12,13,16]*.* Furthermore, Katsuyama *et al.* reported the production of the methylated resveratrols pinostilbene and pterostilbene in recombinant *E. coli* using the pinosylvin methyltransferase (OsPMT) gene from *Oryza sativa* and the tyrosine feeding method in a culture medium [15].

In this study, we describe the production of the methylated resveratrol analogs of pinostilbene, 3,5-dihydroxy-4’-methoxystilbene, 3,4’-dimethoxy-5-hydroxystilbene, and 3,5,4’-trimethoxystilbene using the recombinant *E. coli* that harbors an artificial biosynthetic pathway. These artificial biosynthetic pathways contain a series of codon-optimized *sbOMT1* or *sbOMT3 O*-methyltransferase genes, and both *O*-methyltransferase genes from sorghum in addition to the resveratrol biosynthetic gene cluster. The recombinant *E. coli* produces methylated resveratrol analogs beginning with the simple sugar fermentation, but not in the bioconversion of the intermediates.

## Results and discussion

### Characterization of the resveratrol *O*-methyltransferase via a bioconversion experiment in recombinant *E. coli*

Previous reports have noted that the two resveratrol *O*-methyltransferase genes (*sbOMT1* and *sbOMT3*) from *Sorghum bicolor* are capable of using resveratrol as a substrate that yields methylated analogs of resveratrol [12,13]*.* It was claimed that the *sbOMT3 O*-methyltransferase catalyzes the A-ring specific 3,5-*bis-O*-methylation of resveratrol, which in turn yields pterostilbene (3,5-dimethoxy-4’-hydroxystilbene) in the co-expression system of *sbOMT3* with a stilbene synthase from peanuts (AhSTS3) [13]. In addition, *sbOMT1*, which had previously been identified as a potential eugenol *O*-methyltransferase, predominantly catalyzes the resveratrol B-ring (4’-*O*-methylation), which yields 3,5-dihydroxy-4’-methoxystilbene [12,13].

**Figure 2:**
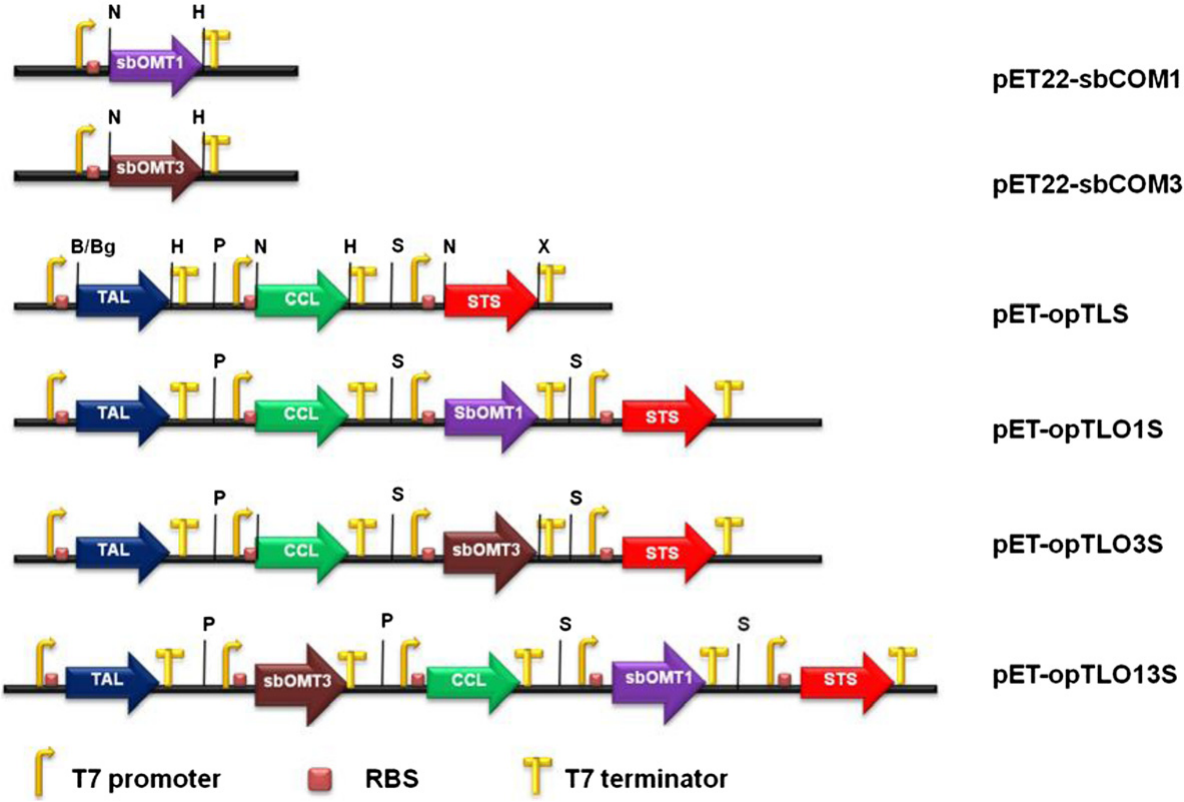
**Expression vectors of *O*-methyltransferase and organization of the artificial gene clusters used for the production of each methylated analogs of resveratrol in *E. coli*.** All constructs contained the T7 promoter, RBS in front of each gene, and T7 terminator at the rear of each gene. B, *Bam*HI; Bg, *Bgl*II; H, *Hind*III; P, *Pac*I; S, *Spe*I; N, *Nde*I; X, *Xho*I.

**Figure 3:**
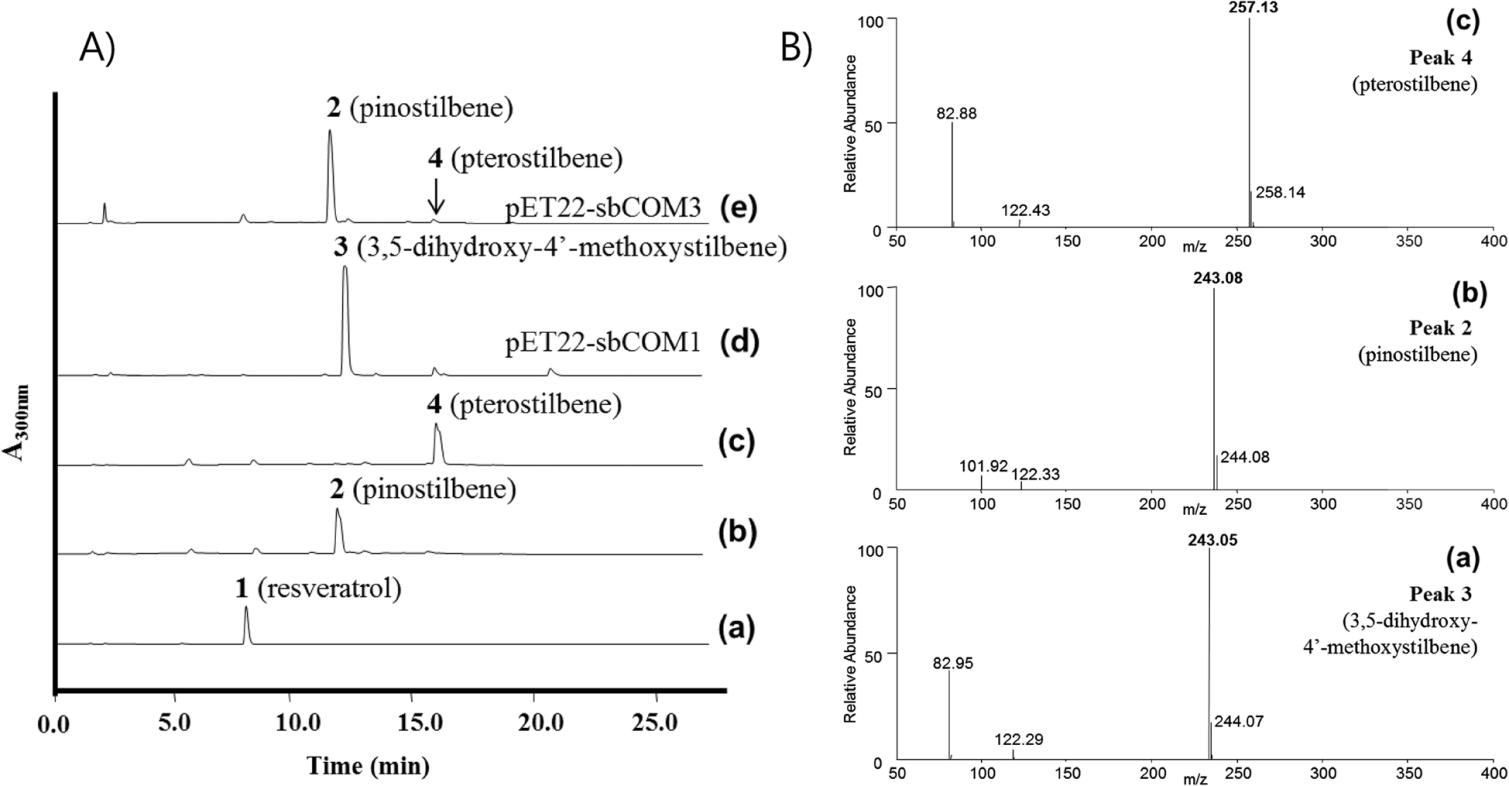
**HPLC profile (A) and selected mass ion chromatogram (B) of bioconversion experiments. A**; HPLC profile of the **(a)** standard resveratrol, **(b)** pinostilbene, and **(c)** pterostilbene; **(d)** resveratrol supplemented *E. coli* harboring pET22-sbCOM1; **(e)** resveratrol supplemented *E. coli* harboring pET22-sbCOM3. The absorbance was monitored at 300 nm. Peak 1, resveratrol; peak 2, pinostilbene; peak 3, 3,5-dihydroxy-4’-methoxystilbene; peak 4, pterostilbene. **B**; Selected mass ion chromatogram of **(a)** 3,5-dihydroxy-4’-methoxystilbene (*m/z* 243.05 [M + H]^+^) produced by *E. coli* harboring pET22-sbCOM1; **(b)** pinostilbene (*m/z* 243.08 [M + H]^+^) and **(c)** pterostilbene (*m/z* 257.13 [M + H]^+^) produced by *E. coli* harboring pET22-sbCOM3. The minor peak of 15.9 min in the HPLC profile **(d)** was not seen in the mass ion peaks of pterostilbene.

In this study, the functions of the codon-optimized resveratrol *O*-methyltransferase genes (*sbOMT1* and *sbOMT3*) were re-evaluated using a bioconversion experiment with resveratrol in recombinant *E. coli*. The codon-optimized synthetic *sbOMT1* and *sbOMT3* genes were cloned in the expression vector pET-22b(+) on the *Nde*I/*Hind*III sites (pET22-sbCOM1 and pET22-sbCOM3, respectively; Figure 2). Each construct was transformed in the *E. coli* C41 (DE3) cells, and the recombinant *E. coli* was selected based on the expression analysis using SDS-PAGE (Additional file 1: Figure S1). In order to investigate whether the recombinant *O*-methyltransferases can catalyze the production of methylated resveratrol derivatives, resveratrol was added to the cultured recombinant *E. coli* harboring pET22-sbCOM1 and pET22-sbCOM3. The culture broth and bacterial cells were collected after 36 hours and were then subjected to HPLC and LC/MS analyses (Figure 3). Under the bioconversion conditions employed in this study most of the feeding resveratrol disappeared and each methylated form was detected as a main peak in the HPLC (Figure 3A). However, when the bioconversion rate was calculated based on a quantitative comparison with feeding substrates and the products, the recombinant *E. coli* harboring pET22-sbCOM1 and pET22-sbCOM3 showed roughly 42% and 12% conversion ratios, respectively (data not shown). These bioconversion ratios are consistent with results recently reported by Jeong *et al.*[16]. It is presumed that a significant amount of additive resveratrol was decomposed in the *E. coli* culture medium.

The recombinant *E. coli* cells that harbored the pET22-sbCOM1 plasmid produced a 12.2 min retention time peak, which is a slightly later retention time than the pinostilbene (3,4’-dihydroxy-5-methoxystilbene) as seen in Figure 3A(d). The major peak in Figure 3A(d) exhibited parent mass ion peaks at *m/z* 243.05 [M + H]^+^, which corresponded to one methylation of resveratrol (an addition of 14 Da; Figure 3B(a)). It was expected that this methylated compound could have a methoxy group located in the 4’ position in the B-ring (Figure 1). In the pET22-sbCOM3 clone, a major peak was present at the same retention time (11.6 min) as that in the HPLC analysis and at the same mass ion peaks at *m/z* 243.08 [M + H]^+^ with authentic pinostilbene, which is one methylation of resveratrol (Figure 3B(b)). However, this recombinant produced a very low level minor peak of 15.9 min in the HPLC, which was the same retention time as the pterostilbene (3,5-dimethoxy-4’-hydroxystilbene; Figure 3A(e)). This minor peak was also accepted based on the mass spectra as *m/z* 257.13 [M + H]^+^, which corresponded to two methylations of resveratrol (an addition of 28 Da; Figure 3B(c)). However, the peak intensity was not sufficient to allow a detailed structural characterization. These results exhibit a controversial conclusion that contrasts with the results reported previously by Rimando *et al*., who demonstrated that the sbOMT3 produced pterostilbene as a major product [13]. However, our results agreed with Jeong *et al*.’s reported results with the *in vitro* and bioconversion activity of the sbOMT3 [16].

### Construction of artificial biosynthetic pathways for the production of methylated resveratrol analogs in *E. coli*

We have previously produced resveratrol in *E. coli* harboring an artificial biosynthetic gene cluster in which the TAL from *Saccharothrix espanaensis*, CCL from *Streptomyces coelicolor*, and STS from the peanut plant *Arachis hypogaea* were contained [18]. In order to produce the methylated resveratrol analogs in *E. coli* using a simple sugar medium, a series of plasmids containing the artificial biosynthetic pathway were constructed (Figure 2). The artificial resveratrol and methylated resveratrol biosynthetic plasmids were constructed using the previously described cloning methods [18], and each plasmid contained genes with their own T7 promoter, ribosome-binding site (RBS), and terminator sequence as in the parental vectors. In this study, a new resveratrol-producing construct of pET-opTLS, which contained codon-optimized *tal* and *sts* genes, and cloned *ccl* gene from *S. coelicolor*, was constructed using previously reported cloning method of the parental vector pET-TLkS [18]. The *E. coli* cells with the pET-opTLS clone exhibited a higher production yield of resveratrol (5.2 mg/L) in the culture system with a modified M9 medium compared with the original pET-TLkS clone (1.4 mg/L) [18]. The cause of this improvement remains unknown, but it is possible that these protein expression ratios may have better optimized the resveratrol production than the original combination.

In order to construct an expression vector that contains the additional *O*-methyltransferase gene(s) that are under the control of the T7 promoter, the DNA fragment containing the promoter, *O*-methyltransferase coding region, and terminator using pET22-sbCOM1 and pET22-sbCOM3 plasmids as templates were amplified. Then, the amplified fragments were inserted into pET-opTLS, which resulted in pET-opTLO1S and pET-opTLO3S, respectively. Similarly, in order to construct the plasmid containing the two *O*-methyltransferases biosynthetic pathways, the *O*-methyltransferase fragment containing the *sbOMT3* coding region was inserted into the pET-opTLO1S, which resulted in pET-opTLO13S. This is useful assembly method for the reconstruction of multi-enzyme biosynthetic pathways such as stilbenes and flavones biosynthesis.

**Figure 4:**
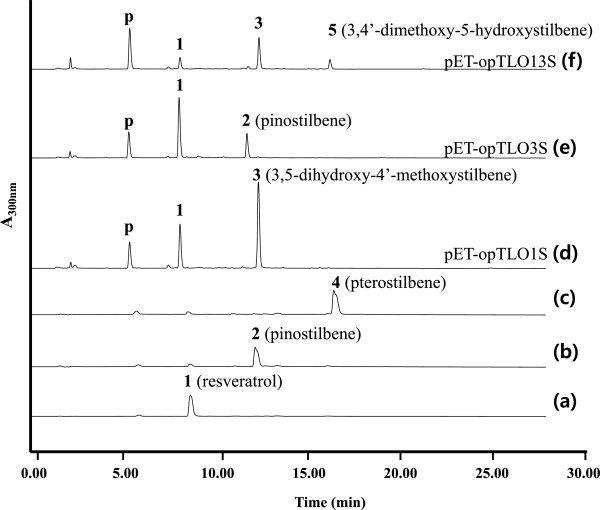
**HPLC profile of methylated resveratrol analogs in recombinant *E. coli*.** HPLC profile of **(a)** the standard resveratrol, **(b)** pinostilbene, and **(c)** pterostilbene; **(d)** culture extract of *E. coli* harboring pET-opTLO1S; **(e)** culture extract of *E. coli* harboring pET-opTLO3S; **(f)** culture extract of *E. coli* harboring pET-opTLO13S in M9 medium. The absorbance was monitored at 300 nm. p, 4-coumaric acid; peak 1, resveratrol; peak 2, pinostilbene; peak 3, 3,5-dihydroxy-4’-methoxystilbene; peak 4, pterostilbene; peak 5, 3,4’-dimethoxy-5-hydroxystilbene.

**Figure 5:**
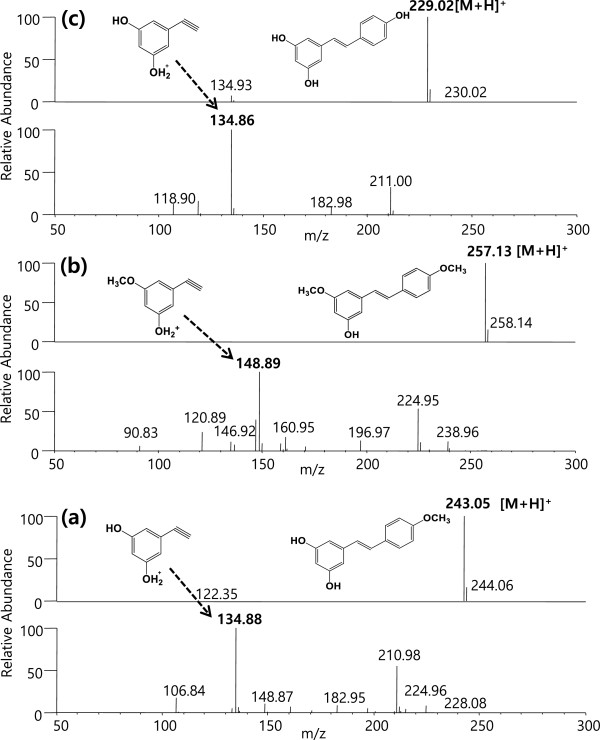
**Mass fragmentation analysis of methylated resveratrol analogs in recombinant *E. coli.*** Structures and MS^n^ spectra of the predicted analogs produced using the artificial biosynthetic pathways. **(a)** 3,5-dihydroxy-4’-methoxystilbene; **(b)** 3,4’-dimethoxy-5-hydroxystilbene; and **(c)** standard resveratrol.

The recombination cells that harbor the artificial biosynthetic gene cluster were cultured in a modified M9 medium. A comparison of the fermentation products of the *E. coli* cells that harbored pET-opTLS, pET-opTLO1S, pET-opTLO3S, and pET-opTLO13S revealed that new peaks were detected in the engineered strain (Figure 4). The retention times for peaks 1 and 2, which were produced in the recombinant pET-opTLO3S, were identical to those of the original resveratrol and pinostilbene, respectively (Figure 4(e)). These peaks were further analyzed using LC/MS/MS in the positive ion mode. Using the LC/MS analysis, peak 1 (resveratrol) and peak 2 (pinostilbene) were identified as *m/z* 229.02 [M + H]^+^ and *m/z* 243.08 [M + H]^+^, respectively, when comparing the obtained fragmentation pattern with that of the original standards. Furthermore, peak 3, which was produced in the recombinant pET-opTLO1S and pET-opTLO13S, was identified as *m/z* 243.05 [M + H]^+^ using the LC/MS. However, the MS/MS analysis of peak 3 exhibited different fragments in the ion pattern with the pinostilbene, and the peaks of *m/z* 134.88 [M + H]^+^ that represent the two hydroxyl group located in the A-ring was also identified (Figure 5(a)). The fragment ion at *m/z* 134.88 [M + H]^+^, which contained the phenolic A-ring of a compound with a triple bond, has already been reported as a distinctive mass fragment of resveratrol [26]. The A-ring cleavage of the resveratrol produced a mass peak of a triple bond (A-ring) at *m/z* 134.86 [M + H]^+^ due to the hydroxy group at the C-3 and C-5 positions (Figure 5(c)). However, due to the presence of a methoxy group in the A-ring in pinostilbene, the triple bond mass peak appeared at *m/z* 148.89 [M + H]^+^. In addition, peak 5 exhibited a very similar retention time and the same mass peak (*m/z* 257.13 [M + H]^+^) as pterostilbene (Additional file 1: Figure S2); however, the major daughter ion peaks of *m/z* 148.89 [M + H]^+^ represented one methoxy group located in the A-ring (Figure 5(b)). Interestingly, the mass fragmentation pattern of pterostilbene, which was found in a detectable amount in the bioconversion experiment with the recombinant pET22-sbCOM3 as mentioned earlier, was not detected in the recombinant pET-opTLO3S and pET-opTLO13S. These results support that peak 5 was not pterostilbene (3,5-dimethoxy-4’-hydroxystilbene), but rather that it was 3,4’-dimethoxy-5-hydroxystilbene, which has methoxy groups located in both the A-ring and B-ring.

### Production and purification of methylated resveratrol analogs in *E. coli* via artificial biosynthetic pathways

**Figure 6:**
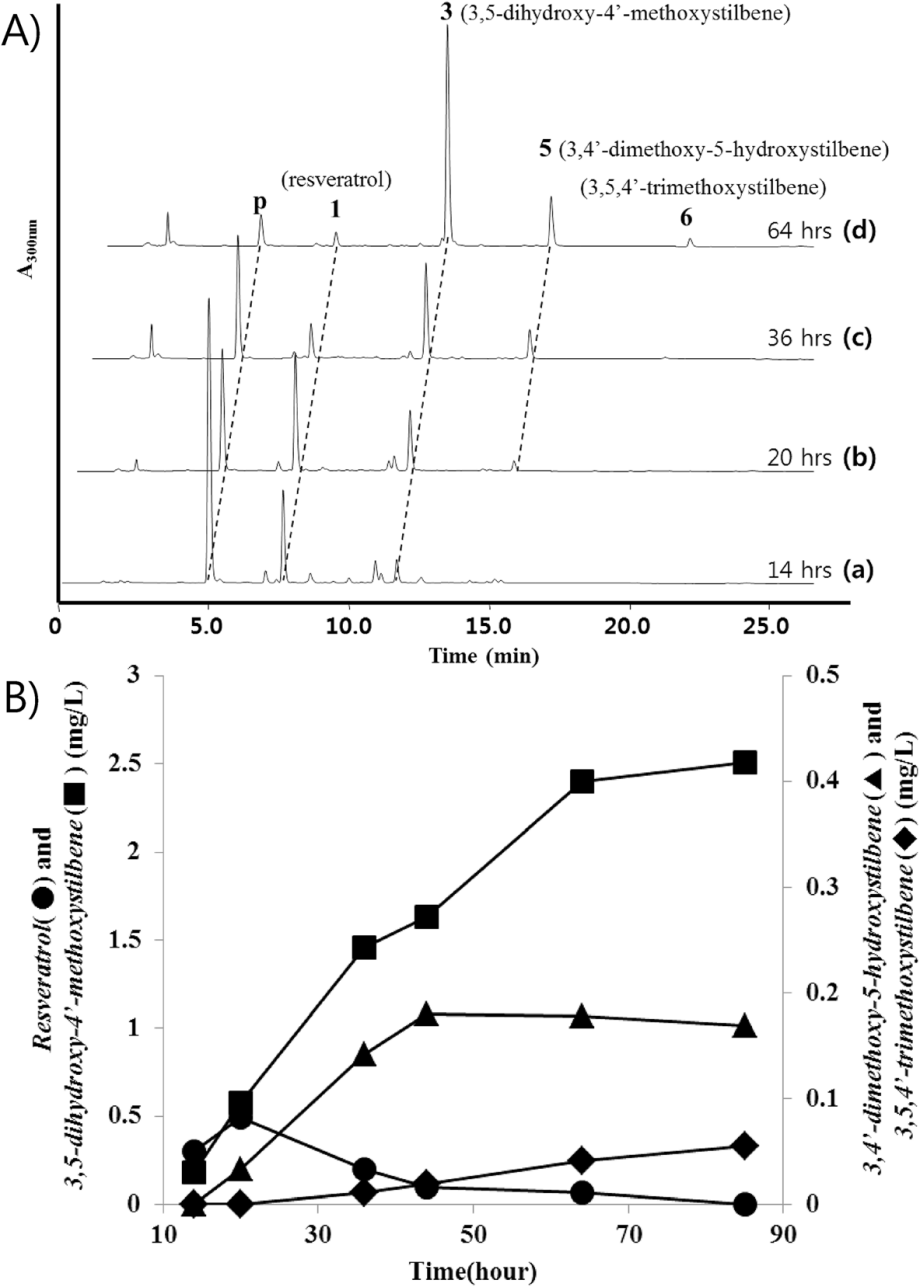
**Accumulation of 3,4’-dimethoxy-5-hydroxystilbene and 3,5,4’-trimethoxystilbene in the culture of *E. coli *harboring pET-opTLO13S. A**. HPLC profile of the time-course cultivation (14, 20, 36, and 64 hours) of *E. coli* harboring pET-opTLO13S. The cell was cultured in modified M9 medium at 26°C for **(a)**, 14 hours; **(b)**, 20 hours; **(c)**, 36 hours; **(d)**, 64 hours. p, 4-coumaric acid; peak 1, resveratrol; peak 3, 3,5-dihydroxy-4’-methoxystilbene; peak 5, 3,4’-dimethoxy-5-hydroxystilbene; peak 6, 3,5,4’-trimethoxystilbene. **B**. Production pattern of one, *bis*, and *tri*-methylated resveratrol in the each culture time. ●, resveratrol; ■, 3,5-dihydroxy-4’-methoxystilbene; ♦, 3,4’-dimethoxy-5-hydroxystilbene; ♦. 3,5,4’-trimethoxystilbene. The concentrations were determined through the use of the corresponding chemical standards. All experiments were performed in triplicates.

The 3,4’-dimethoxy-5-hydroxystilbene synthesis levels in the *E. coli* cells that harbored pET-opTLO13S were not sufficient for structure elucidation. For a higher production of the *bis*-methylated resveratrol, the recombinant *E. coli* cells that harbored pET-opTLO13S were investigated using metabolite pattern analyses according to the culture times for 85 hours (Figure 6). This recombinant strain produced a relatively large amount of 3,5-dihydroxy-4’-methoxystilbene from the early stages of the culture. However, at the late stage of the culture, the resveratrol was consumed and the production of methylated resveratrol analogs slightly increased. The production level of 3,4’-dimethoxy-5-hydroxystilbene (peak 5) increased following the culturing and a new peak (peak 6) was also detected that exhibited a more delayed retention time (Figure 6A). The new peak was identified via LC/MS (*m/z* 271.14 [M + H]^+^) and it corresponded to the three methylations of resveratrol (an addition of 42 Da; Additional file 1: Figure S3). This result represents the methoxy moiety located at the C-3 and C-5 positions in the A-ring and the C-4’ position in the B-ring (Figure 1).

In order to obtain NMR-accessible amounts from the present culture conditions, it was scaled up to 4 liters of *E. coli* fermentation that harbored pET-opTLO13S. Three methylated resveratrol analogs were purified from the large culture broth. The mass spectra of the purified methylated resveratrol analogs (3,5-dihydroxy-4’-methoxystilbene, 3,4’-dimethoxy-5-hydroxystilbene, and 3,5,4’-trimethoxystilbene) are characterized with mass peaks at *m/z* 243, 257, and 271 [M + H]^+^, respectively. The ^1^H NMR spectra of the purified compounds were similar to those of the one, *bi*, and *tri*-methyl analogs that were isolated, respectively (Additional file 2: Table S1). The structures of the purified resveratrol analogs were identified through spectral data interpretation and comparison with the values reported in the literature [27]. The coexistence of sbOMT1 and sbOMT3 produced a relatively large amount of 3,5-dihydroxy-4’-methoxystilbene from the early stage of the culture; however, the pinostilbene was not significantly detected (Figure 6). These results indicate that sbOMT1 has superior methylation activity against resveratrol compared with that of sbOMT3. However, at the late stage of the culture, the production yield of 3,4’-dimethoxy-5-hydroxystilbene increased; it finally produced *tri*-methyl resveratrol (3,5,4’-trimethoxystilbene) (Figure 6B). Therefore, 3,4’-dimethoxy-5-hydroxystilbene is a major *bis*-methylated intermediate in the biosynthesis of 3,5,4’-trimethoxystilbene in this *E. coli* system. These results indicate that sbOMT3 has methylation activity in the A ring hydroxyl group against resveratrol as well as 3,5-dihydroxy-4’-methoxystilbene. Furthermore, the presence of 3,5,4’-trimethoxystilbene without the detection of pterostilbene indicates that the C-5 methylation is expected to be the final modification step in the coexistence of the sbOMT1 and sbOMT3 enzymes.

Although the production yield of these compounds is too low for commercial purposes, the results presented here were obtained using the wild type *E. coil*. This is the first report of an artificial biosynthetic pathway to obtain methylated resveratrol compounds used a single vector system joined with resveratrol biosynthetic genes and two resveratrol *O*-methyltransferase genes. The recombinant *E. coli* produces methylated resveratrol analogs beginning with simple fermentation rather than with bioconversion of intermediates. Recently, large-scale production of plant metabolites via metabolic engineered microbes has provided a promising alternative to chemical synthesis and extraction from raw plant sources [28,29]. Further application with a metabolically optimized host strain, i.e. a tyrosine and/or malonyl CoA high producer [23,24], may be very useful for obtaining industrial scale production yields.

## Conclusions

We developed new artificial biosynthetic pathway for methylated resveratrol compounds using a single vector system joined with resveratrol biosynthetic genes and sorghum two resveratrol *O*-methyltransferase genes. An *E. coli* system containing an artificial biosynthetic pathway produced methylated resveratrol analogs of pinostilbene, 3,5-dihydroxy-4’-methoxystilbene, 3,4’-dimethoxy-5-hydroxystilbene, and 3,5,4’-trimethoxystilbene from a simple sugar medium without any precursor feeding. The *E. coli* cells that harbored the pET-opTLO1S plasmid (combined with *O*-methyltransferase (sbOMT1) and a resveratrol biosynthetic pathway) produced the one-methyl resveratrol analog, 3,5-dihydroxy-4’-methoxystilbene. The *E. coli* cells that harbored the pET-opTLO3S plasmid (combined with *O*-methyltransferase (sbOMT3) and a resveratrol biosynthetic pathway) produced the other one-methyl resveratrol analog, pinostilbene. Furthermore, the *E. coli* cells that harbored the pET-opTLO13S (combined with two sbOMT1 and sbOMT3 *O*-methyltransferases and a resveratrol biosynthetic pathway) produced 3,5-dihydroxy-4’-methoxystilbene, a *bis*-methyl resveratrol (3,4’-dimethoxy-5-hydroxystilbene), and a *tri*-methyl resveratrol (3,5,4’-trimethoxystilbene) (Figure 1). The coexistence of sbOMT1 and sbOMT3 produced 3,5-dihydroxy-4’-methoxystilbene as a major product in the early stage of the culture; however, the pinostilbene was not significantly detected (Figure 6). Katsuyama *et al.* reported the production of the pinostilbene and pterostilbene from *E. coli* culture using the fungal *O*-methyltransferase (OsPMT) gene and the tyrosine feeding medium [15]. Our described strategy exhibits the first harnessing of microorganisms for the *de novo* synthesis of methylated resveratrol analogs using a one-vector system from a simple sugar without precursor feeding in the culture medium. Thus, this is also the first report on the production of the methylated resveratrol compounds *bis*-methyl and *tri*-methyl resveratrol (3,4’-dimethoxy-5-hydroxystilbene and 3,5,4’-trimethoxystilbene) in the *E. coli* culture. These results provide an opportunity to create an economic incentive to develop strains capable of converting cheaper feedstock into high value compounds.

## Methods

### Bacterial strains, plasmids, and chemicals

*E. coli* DH5α and *E. coli* C41 (DE3) [30] were used in the general DNA manipulation and expression of biosynthetic genes, respectively. T-blunt vector (Solgent, Korea) was used in the polymerase chain reaction (PCR) cloning. pET-22b(+) and pET-28a(+) were purchased from Novagen (USA). 4-coumaric acid, resveratrol, pterostilbene, and 3,5,4’-trimethoxystilbene were purchased from Sigma-Aldrich (USA). Pinostilbene was purchased from Tokyo Chemical Industry, Co (Japan). IPTG was purchased from A.G. Scientific, Inc (USA). NMR solvent was purchased from Cambridge Isotope Laboratories, Inc. (USA). Hypergrade solvent for LC-MS was purchased from Merck KGaA (Germany).

### DNA manipulation

The restriction enzymes (NEB, Takara), Ex taq polymerase (Takara) pfu taq polymerase (Solgent), and an AccuPower Ligation kit (Bioneer, Korea) were used according to the instructions provided by the manufacturers. The codon optimization and synthesis of *tal* gene from *Saccharothrix espanaensis* (KCTC9392) and the *sbOMT1 & sbOMT3* genes [GenBank:ABP01563.1, ABP01564.1] from *Sorghum bicolor* were performed using the GeneGPS™ program (DNA2.0). The *sts* gene from *Arachis hypogaea* [GenBank: AB027606] was codon-optimized and synthesized using Codon Devices (USA). The *ccl* gene from *Streptomyces coelicolor* A3(2) [GenBank: NP628552] was cloned and characterized in the laboratory. After the DNA manipulation, the absence of undesired alterations during the PCR was verified using nucleotide sequencing on an automated nucleotide sequencer.

### Construction of O-methyltransferase expression vectors and assembly of the artificial biosynthetic pathways

The list of plasmids and strains used in this study can be found in Table 1. The plasmids were assembled using a serial stepwise cloning process as previously reported [18,31]. Briefly, the five genes (*TAL*, *CCL, STS, sbOMT1*, and *sbOMT3*) were independently cloned into pET-22b(+) or pET-28a(+) vectors. In order to construct an expression vector containing the *O*-methyltransferase genes that were under the control of independent T7 promoters, the 1.13-kb and 1.12-kb DNA fragments, which contain the sbOMT1 and sbOMT3 coding regions, were synthesized and then cloned into the *Nde*I and *Hind*III sites on pET-22b(+), which resulted in pET22-sbCOM1 and pET22-sbCOM3, respectively. In order to assemble the vector containing the methylated resveratrol artificial biosynthetic pathways, the sbOMT1 and sbOMT3 coding regions were amplified using pET22-sbCOM1 and pET22-sbCOM3, respectively, as templates with the primer NSpe (the sequence is located upstream of the T7 promoter region of the pET vector and contains the designed *Spe*I site: ACTAGTAGGTTGAGGCCGTTGAGCACCGCC) and CSpe (the sequence is located downstream of the T7 terminator region of the pET vector and contains the designed *Spe*I site: ACTAGTTCCTCCTTTCAGCAAAAAACCCCTC). Each of the amplified fragments were digested with *Spe*I and cloned between the *Spe*I digested pET-opTLS via ligation, which resulted in pET-opTLO1S and pET-opTLO3S. In order to construct the pET-opTLO13S vectors, the 2.1-kb DNA fragment containing the sbOMT3 coding region was PCR-amplified with the NPac (the sequence is located upstream of the T7 promoter region of the pET vector and contains the designed *Pac*I site: TTAATTAATCGCCGCGACAATTTGCGACGG) and CPac (the sequence is located downstream of the T7 terminator region of the pET vector and contains the designed *Pac*I site: TTAATTAATGCGCCGCTACAGGGCGCGTCC) primers, respectively, using pET22-sbCOM3 as a template. The amplified fragment was digested with *Pac*I and cloned between the *Pac*I digested pET-opTLO1S, which resulted in pET-opTLO13S. The gene sequences and orientations were verified via sequencing after each round of cloning.

**Table 1:** Plasmids and strains used in this study

**Plasmid or strain**	**Relevant characteristics**	**Source**
Plasmids		
pET-22b(+)	f1 ori, T7 promoter, Amp^R^	Novagen
pET-28a(+)	f1 ori, T7 promoter, Kan^R^	Novagen
pET-opTAL	pET-28a(+) carrying codon-optimized *Saccharothrix espanaensis* TAL	Kang *et al.*[31]
pET-CCL	pET-28a(+) carrying CCL from *Streptomyces coelicolor*	Choi *et al.*[18]
pET-KSTS	pET-28a(+) carrying codon-optimized *Arachis hypogaea* STS	Choi *et al.*[18]
pET22-sbCOM1	pET-22b(+) carrying codon-optimized *Sorghum bicolor* sbOMT1	This study
pET22-sbCOM3	pET-22b(+) carrying codon-optimized *Sorghum bicolor* sbOMT3	This study
pET-opTLS	pET-28a(+) carrying opTAL, CCL, and KSTS	This study
pET-opTLO1S	pET-28a(+) carrying opTAL, CCL, KSTS, and sbCOM1	This study
pET-opTLO3S	pET-28a(+) carrying opTAL, CCL, KSTS, and sbCOM3	This study
pET-opTLO13S	pET-28a(+) carrying opTAL, CCL, KSTS, sbCOM1, and sbCOM3	This study
Stranis		
*E. coli* DH5a	cloning host	Invitrogen
*E. coli* C41(DE3)	derivative strain of *E. coli* BL21(DE3)	Miroux B & Walker JE [30]

### Production of methylated resveratrol analogs

The recombinant *E. coli* C41 (DE3) strains that harbored plasmids were precultured overnight at 37°C in a *Luria-Bertani* (LB) medium containing 50 μg/mL of appropriate antibiotics (ampicillin for to maintain the pET-22b(+)-derived plasmids and kanamycin for the pET-28a(+)-derived plasmids). The overnight culture was inoculated (1.5%) into a fresh LB medium supplemented with the same concentration of appropriate antibiotics. The culture was grown at 37°C to an optical density of 600 nm (OD_600_) with 0.6. IPTG being added to the final concentration of 1 mM, and the culture was incubated for 6 hours. The cells were harvested via centrifugation, and then suspended and incubated at 26°C for 36 hours in a modified M9 medium (M9 medium supplemented with 15 g/L glucose, 25 g/L CaCO_3_, 1 mM IPTG and 50 μg/mL appropriate antibiotics). For the feeding experiments, the cultures were supplemented with resveratrol (final concentration: 70 μM) and allowed to grow for an additional 36 hours.

Twenty milliliters of culture was extracted with an equal volume of ethyl acetate. The ethyl acetate was dried and resuspended in 200 μL of methanol. Twenty microliters of the extract was applied to a J’sphere ODS-H80 column (4.6 × 150 mm i.d., 5 μm; YMC, Japan) using a high-performance liquid chromatography (HPLC) system [CH_3_CN-H_2_O (0.05% trifluoroacetic acid(TFA)) 20% to 100% acetonitrile (CH_3_CN) for 25 min, 100% CH_3_CN for 5 min, at flow rate of 1 mL/min; Dionex, USA] equipped with a photodiode array detector. A liquid chromatography-mass spectrometry (LC-MS) was performed using an LTQ XL linear ion trap (Thermo Scientific, USA) equipped with an electrospray ionization (ESI) source that was coupled to a rapid separation LC (RSLC; ultimate 3000, Thermo Scientific) system (ESI-LC–MS) using a Cosmosil 2.5 Cholester column (Nacalai Tesque, Japan) (2.0 × 100 mm; 2.5 μm particle size) with a linear gradient of the binary solvent system under the same HPLC conditions as described above. The ESI (positive ion) parameters for the methylated resveratrol compounds were the source voltage (+5KV), entrance capillary voltage (+18 V) entrance capillary temperature (275°C), and tube lens voltage (+120 V). The scan range was fixed from m/z 50 to 1000. The data-dependent mass spectrometry experiments were controlled using the menu driven software provide with the Xcalibur system (version 2.2 SP1.48; Thermo Scientific). The compounds were identified through comparisons with the standard compounds using the observed retention time, ultraviolet spectra, and mass chromatogram.

### Purification and structural elucidation of the methylated resveratrol analogs

The recombinant *E. coli* strains that harbored the pET-opTLO13S plasmid were cultured via the same method as described earlier, but the culture volume was increased to 4 liters. The isolation processes were undertaken as described above. The EtOAc-soluble material (0.9 g) was further purified by reverse-phase HPLC (Waters Co., USA) using the YMC J’sphere ODS-H80 (10 × 250 mm, 3 mL/min) with a linear gradient from 20% to 100% CH_3_CN containing 0.05% TFA in order to yield 3,5-dihydroxy-4’-methoxystilbene (4.2 mg), 3,4’-dimethoxy-5-hydroxystilbene (2.1 mg), and 3,5,4’-trimethoxystilbene (0.9 mg). The structural elucidation of the purified compounds was undertaken using ^1^H NMR spectroscopy. The NMR experiments were performed on a Bruker AVANCE 800 spectrometer (800 MHz; Bruker Inc., USA) and Barianunity^Ionva^-400 instrument. The structures of 3,5-dihydroxy-4’-methoxystilbene, 3,4’-dimethoxy-5-hydroxystilbene and 3,5,4’-trimethoxystilbene were determined based on the NMR data.

## Competing interests

The authors declare that they have no competing interests.

## Authors’ contributions

SK and JL performed the experiments and wrote the manuscript. SK, OC, and CY coperformed the experiments on the metabolite analysis, molecular cloning, and bioconversion. JL and CY performed the experiments on the metabolite LC/MS analysis, purification, and structure elucidation. BH and JJ contributed general advice, particularly on the metabolite analysis and resource support. YH designed all the experiments and wrote the manuscript. All authors read and approved the final manuscript.

## Supplementary Material

Additional file 1: Figure S1 SDS-PAGE analysis of the expression of sbOMT1 and sbOMT3 enzymes in *E. coli*. **Figure S2.** Selected mass ion chromatograms of the pterostilbene (*m/z* 257.13). **Figure S3.** Selected mass ion chromatograms of the 3,5,4’-trimethoxystilbene (*m/z* 271.14) produced by *E. coli* harboring pET-opTLO13S. Click here for file

Additional file 2: Table S1 Structure elucidation of methylated resveratrol analogs. 1H NMR spectra of the purified 3,5-dihydroxy-4’-methoxystilbene, 3,4’-dimethoxy-5-hydroxystilbene, and 3,5,4’-trimethoxystilbene and data from the literature for resveratrol. Click here for file
