# Supplementary material for: Biosynthesis of methylated resveratrol analogs through the construction of an artificial biosynthetic pathway in E. coli
Source: BMC Biotechnol. 2014 Jul 17;14:67. doi: 10.1186/1472-6750-14-67 (PMC4118633; doi:10.1186/1472-6750-14-67)
Supplement: Additional file 1: Figure S1 — SDS-PAGE analysis of the expression of sbOMT1 and sbOMT3 enzymes in E. coli. Figure S2. Selected mass ion chromatograms of the pterostilbene (m/z 257.13). Figure S3. Selected mass ion chromatograms of the 3,5,4’-trimethoxystilbene (m/z 271.14) produced by E. coli harboring pET-opTLO13S. [file 1472-6750-14-67-S1.pptx]

## Slide 1
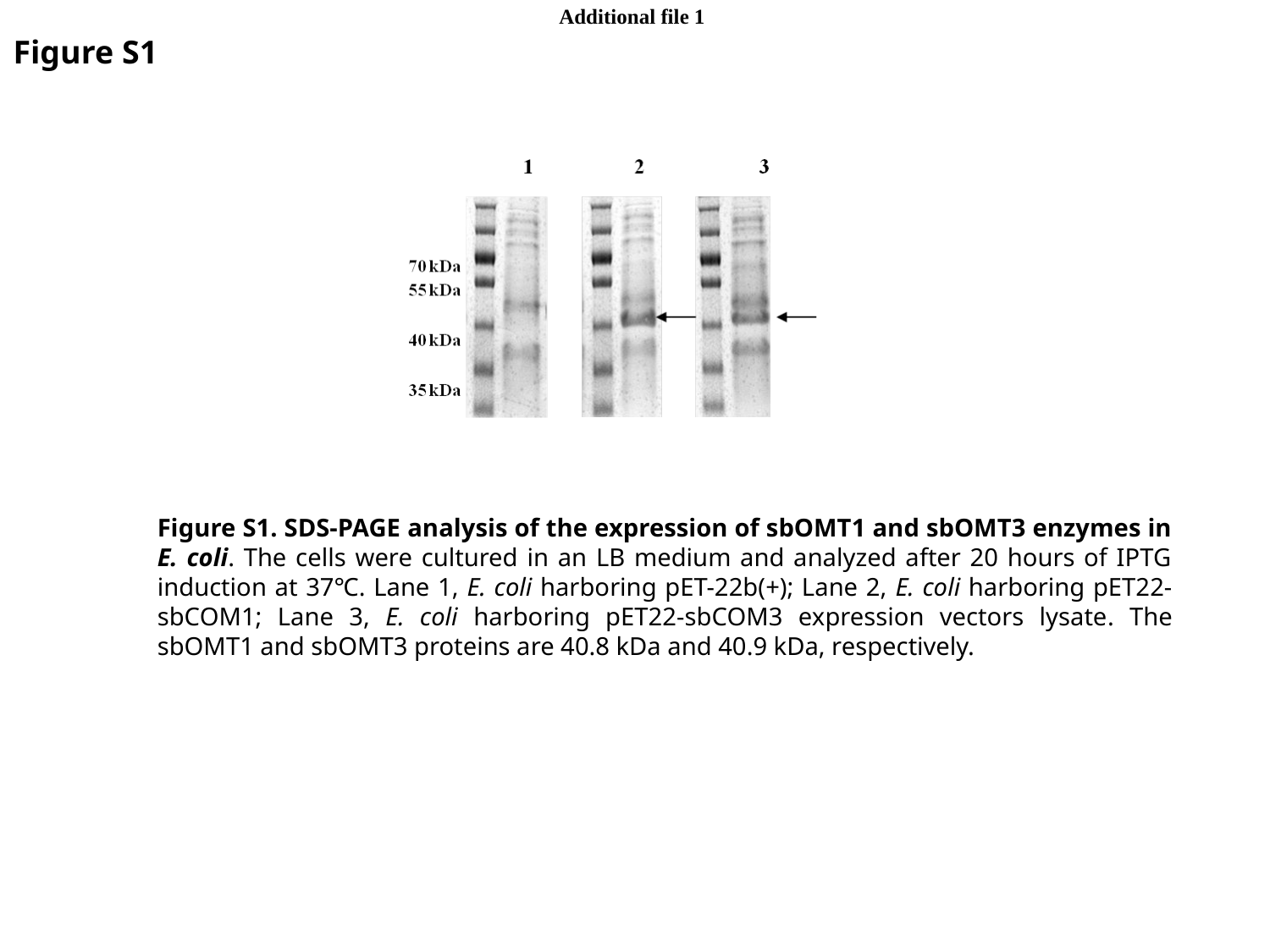

Additional file 1
Figure S1
Figure S1. SDS-PAGE analysis of the expression of sbOMT1 and sbOMT3 enzymes in E. coli. The cells were cultured in an LB medium and analyzed after 20 hours of IPTG induction at 37℃. Lane 1, E. coli harboring pET-22b(+); Lane 2, E. coli harboring pET22-sbCOM1; Lane 3, E. coli harboring pET22-sbCOM3 expression vectors lysate. The sbOMT1 and sbOMT3 proteins are 40.8 kDa and 40.9 kDa, respectively.

## Slide 2
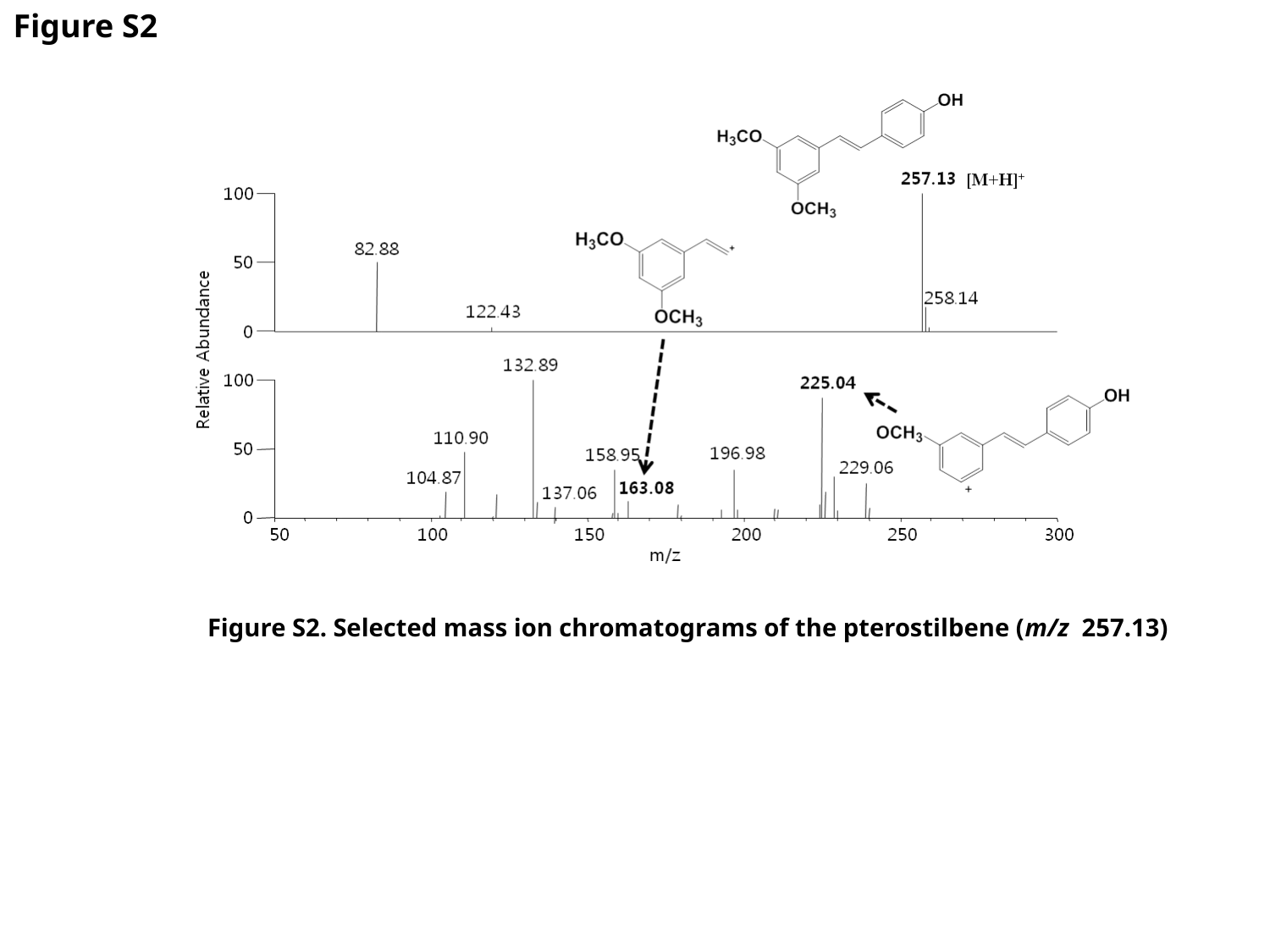

Figure S2
Figure S2. Selected mass ion chromatograms of the pterostilbene (m/z 257.13)

## Slide 3
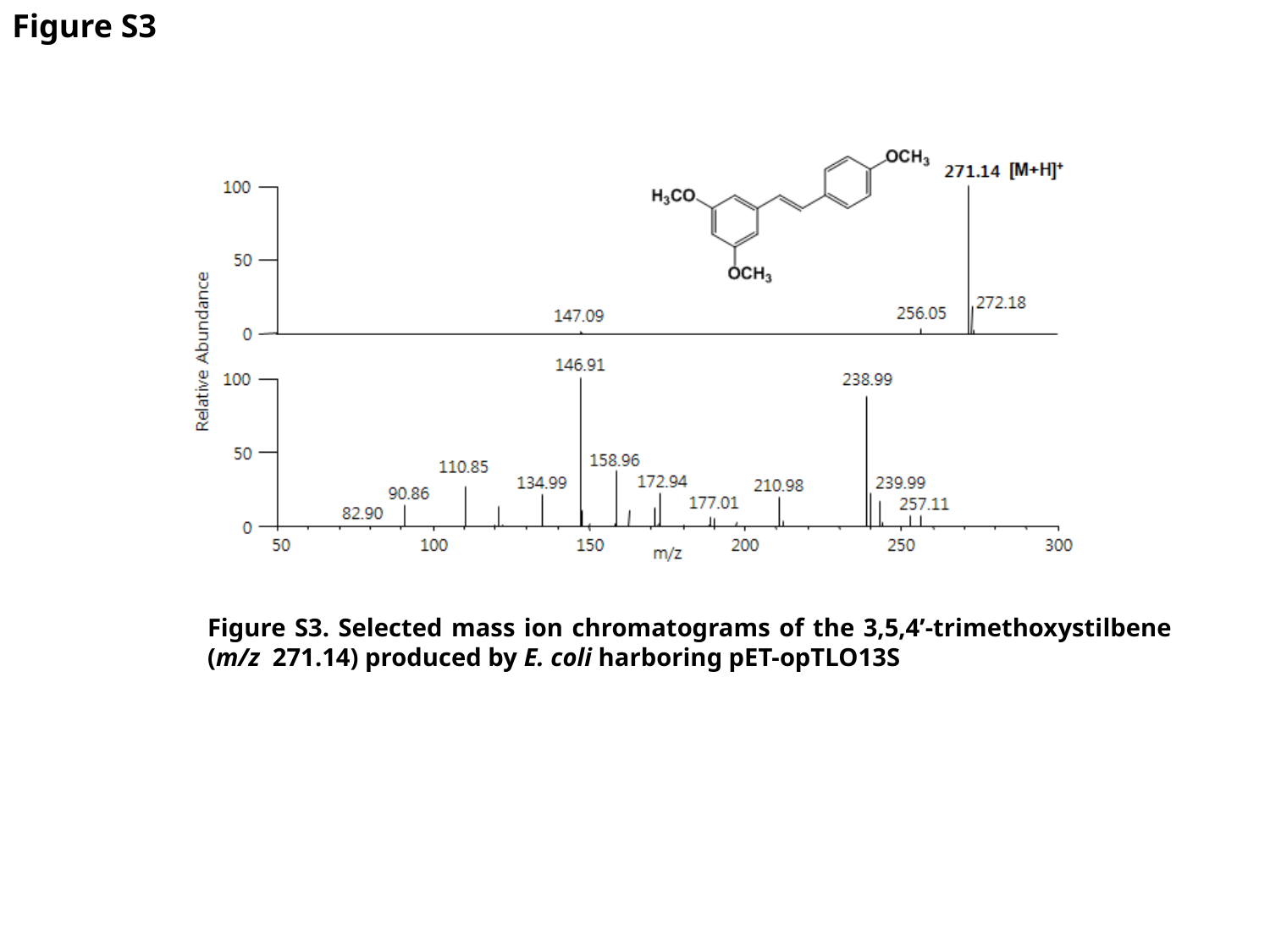

Figure S3
Figure S3. Selected mass ion chromatograms of the 3,5,4’-trimethoxystilbene (m/z 271.14) produced by E. coli harboring pET-opTLO13S
