# Supplementary material for: Biosynthesis of methylated resveratrol analogs through the construction of an artificial biosynthetic pathway in E. coli
Source: BMC Biotechnol. 2014 Jul 17;14:67. doi: 10.1186/1472-6750-14-67 (PMC4118633; doi:10.1186/1472-6750-14-67)

### Additional file 2

### Structure elucidation of methylated resveratrol analogs

From the ^1^H NMR spectra comparison with 3,5-dihydroxy-4’-methoxystilbene and pinostilbene, the upfield shift of the C-4’ signals at δ 3.77 (3H, s, 1-OCH_3_) and the one overlapped phenolic proton of the C-3 and C-5 signal at δ 9.21 (2H, s) indicated that 3,5-dihydroxy-4’-methoxystilbene has more free hydroxyl groups at the C-3 and C-5 on the A-ring than at C-4’ on the B ring, as expected. The recognition of the two methoxy protons at C-3 on the A-ring and C-4’ on the B-ring of 3,4’-dimethoxy-5-hydroxystilbene was confirmed using the separated upfield shift signals at δ3.72 (3H, s, 1-OCH3) and δ3.77 (3H, s, 1-OCH3), respectively. In addition, the presence of three methoxy protons δ3.77 (9H, s, 3-OCH_3_) and disappearance of all phenolic proton signals in 3,5,4’-trimethoxystilbene indicated that the three hydroxyl groups of the resveratrol skeleton were methylated.

## Table S1. ^1^H NMR spectra of the purified 3,5-dihydroxy-4’-methoxystilbene, 3,4’-dimethoxy-5-hydroxystilbene, and 3,5,4’-trimethoxystilbene and data from the literature for resveratrol


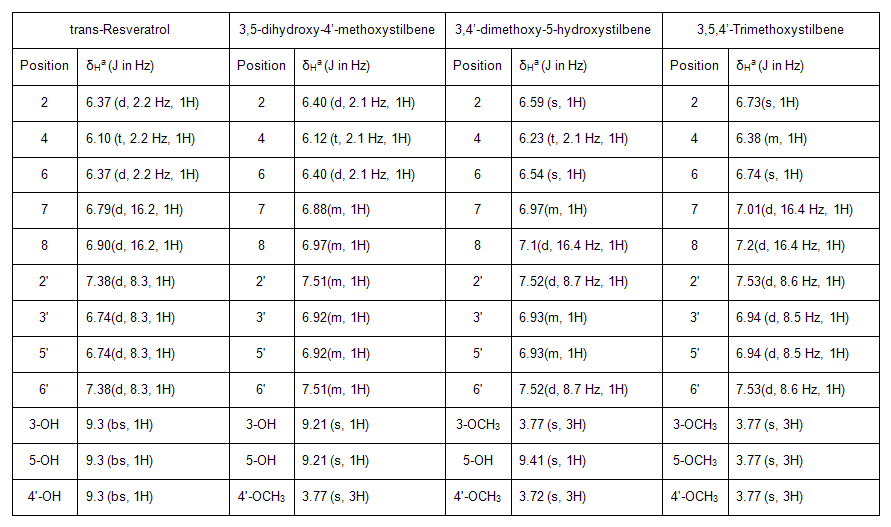

Supplement: Additional file 2: Table S1 — Structure elucidation of methylated resveratrol analogs. 1H NMR spectra of the purified 3,5-dihydroxy-4’-methoxystilbene, 3,4’-dimethoxy-5-hydroxystilbene, and 3,5,4’-trimethoxystilbene and data from the literature for resveratrol. [file 1472-6750-14-67-S2.docx]
